# A survey on 3D object detection in real time for autonomous driving

**DOI:** 10.3389/frobt.2024.1212070

**Published:** 2024-03-06

**Authors:** Marcelo Contreras, Aayush Jain, Neel P. Bhatt, Arunava Banerjee, Ehsan Hashemi

**Affiliations:** ^1^ University of Alberta, Edmonton, AB, Canada; ^2^ Indian Institute of Technology Kharagpur, Kharagpur, West Bengal, India

**Keywords:** 3D object detection, autonomous navigation, visual navigation, robot perception, automated driving systems (ADS), visual-aided decision

## Abstract

This survey reviews advances in 3D object detection approaches for autonomous driving. A brief introduction to 2D object detection is first discussed and drawbacks of the existing methodologies are identified for highly dynamic environments. Subsequently, this paper reviews the state-of-the-art 3D object detection techniques that utilizes monocular and stereo vision for reliable detection in urban settings. Based on depth inference basis, learning schemes, and internal representation, this work presents a method taxonomy of three classes: model-based and geometrically constrained approaches, end-to-end learning methodologies, and hybrid methods. There is highlighted segment for current trend of multi-view detectors as end-to-end methods due to their boosted robustness. Detectors from the last two kinds were specially selected to exploit the autonomous driving context in terms of geometry, scene content and instances distribution. To prove the effectiveness of each method, 3D object detection datasets for autonomous vehicles are described with their unique features, e. g., varying weather conditions, multi-modality, multi camera perspective and their respective metrics associated to different difficulty categories. In addition, we included multi-modal visual datasets, i. e., V2X that may tackle the problems of single-view occlusion. Finally, the current research trends in object detection are summarized, followed by a discussion on possible scope for future research in this domain.

## 1 Introduction

Automated Driving Systems (ADS) and Advanced Driver-Assistance Systems (ADAS) with robust controls are primarily deployed with the intention to reduce human errors in perception and decision-making while enhancing traffic flow and transportation safety in emergency cases and hand-over scenarios (Bengler et al., 2014; Wang et al., 2017; Schwarting et al., 2018; Chen et al., 2019). To this end, ADS represents a significant enhancement in life quality by reducing pollution emissions due to efficiency in construction and driving, travel conformity, and increased productivity that relies on mobility and, consequently, powers regional economies (Greenblatt and Shaheen, 2015; Williams et al., 2020; Silva et al., 2022). On the contrary, it also raised substantial social concerns about policy-making, industry standards, equality of accessibility to unrepresented social groups (i.e., third-world countries, gender or income), insurance costs, and labor-hand reduction with biased accessibility for education to adapt to newer positions (Bissell et al., 2018; Shahedi et al., 2023). Still, the social studies in this matter only covered a narrow spike of the whole problem; researchers claimed a need for a more holistic view due to evidence of only attention in the first two topics (Bissell et al., 2018). Besides, information on light and noise contamination of ADS is sparse and current emissions reports may changed under denser traffic flow after the including of AVs (Silva et al., 2022). We encourage a more profound analysis of the implications of broadly adopting AVs as the primary transport means or inside a hybrid scheme with non-autonomous vehicles. One of the most significant worries about this technology is its security and reliability (Othman, 2021).

ADS can only function safely and effectively if they have access to reliable perception and increased environmental awareness (Hu et al., 2019; Zhang et al., 2022a; Hashemi et al., 2022). In this regard, ADS and their control systems utilize multi-modal sensory data (from stereo or monocular cameras, light detection and ranging (LiDAR), radars, and global navigation satellite systems, GNSS) to 1) achieve semantic information about their surroundings for motion planning 2) identify various static and dynamic objects on the road, pedestrians, etc., 3) estimate their states (e.g., position, heading, and velocity) and 4) to predict trajectories of these objects for safety-critical scenarios (Ji et al., 2018; Marzbani et al., 2019; Mohammadbagher et al., 2020; Bhatt et al., 2022; Bhatt et al., 2023). An unreliable identification of street objects and road signs may lead to catastrophic outcomes and thus, the object detection task is of fundamental importance for safe operation, decision making and controls in autonomous driving (Chen et al., 2021; Gupta et al., 2021). One of the primary reasons behind the failure of object detection in perceptually degraded conditions (i.e., extreme lighting and weather conditions such as snow, hail, ice storms) and adversarial ones is the limitation of sensory data which necessitates multi-modal data fusion (Michaelis et al., 2019; Hnewa and Radha, 2021) Moreover, inconsistency in layout of motorways presents additional complexity for reliable identification of spatial constraints for motion planning in highly dynamic environments; for instance, vehicles in urban areas parked in an arbitrary orientation hinder vehicles from following well-defined driving lanes. Lastly, there always remains a high possibility of occlusion where objects block each other’s view resulting in either partial or complete concealment of the objects. Despite these challenges due to perceptually-degraded conditions and highly dynamic environments, there has been substantial progress in camera-based object detection and state estimation approaches to enhance perception and situational awareness in autonomous driving (Ranft and Stiller, 2016; Arnold et al., 2019; Cui et al., 2019; Gupta et al., 2021) In this regard, visual-based 2D or 3D object detection methodologies in the literature falls into 3 main categories of learning-based, geometrical or model-based, and hybrid approaches. Geometrically constrained model broadly exploit common scenery in AV, e. g., scale inference from road distance to the camera or triangulation between multiple vehicle detections. The hybrid methods aim to fuse the progress made by end-to-end detectors, which are not necessarily designed for AD applications, with the unique features from the first one. Significant research works have been conducted with focus on visual-based 2D object detection (Geiger et al., 2013; Mukhtar et al., 2015; Pendleton et al., 2017; Ku et al., 2018) for autonomous navigation. For object detection in the case of AVs, a conventional pipeline consists of *segmentation* (such as via voxel clustering (Azim and Aycard, 2014) and graph-segmentation methods (Wang et al., 2012)), *feature extraction* using probabilistic feature-based voxels and *classification* based on various state-of-the-art classifiers, such as YOLOv7 (Wang et al., 2022), EfficientDet (Tan et al., 2020) and Swin ViT. Traditional approaches have optimized each of these stages individually (Mousavian et al., 2017; Gählert et al., 2020), while recent end-to-end learning frameworks, which derive a region of interest (ROI) for feature extraction, tend to optimize the whole pipeline (Li et al., 2019; Liu et al., 2019;Liu et al., 2020).

To this end, this work presents an overview of visual-based object detection methods in the context of autonomous driving (AD). The performance of state-of-the-art detection models proposed in the literature is evaluated using popular datasets and well established metrics. This is followed by a thorough review of monocular and stereo camera-based object detection methods. Finally, research gaps and possible directions for future research are identified. The rest of the paper is organized as follows: In Section 2, 2D object detection and challenges are elaborated on; this is followed by a detailed discussion on the recent progress on 3D object detection in Section 4. Finally the future trends and the concluding remarks are summarized in Section 5 and Section 4 respectively.

## 2 Two-dimensional object detection

**FIGURE 1:**
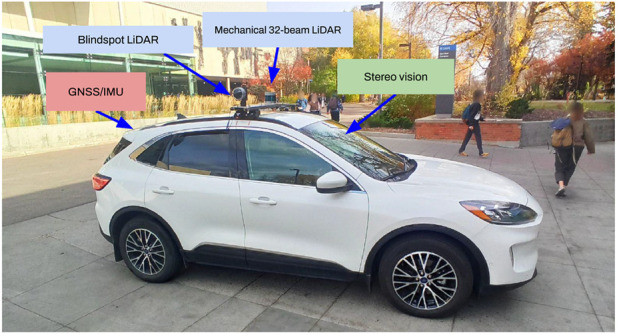
A hybrid electric vehicle at the NODE lab equipped with multi-modal sensors and data fusion systems for perception, motion planning, autonomous navigation, and controls in perceptually-degraded conditions.

Object detection initially started as a classification and instance localization problem in 2D images for automated driving systems which are equipped with multi-modal sensory measurement units (as shown in Figure 1). Detection models employed handcrafted features by Histogram of gradients (HOG), Scale-invariant feature transformation (SIFT), or Oriented fast and rotated BRIEF (ORB) and passed them through a linear classifier, i.e., Support vector machines (SVM). However, with the advent of deep learning methodologies, better solutions which exploited spatial and semantic information under several variances, including scale, translation, and rotation were explored. These algorithms fall into 2 main categories: two-stage detectors and one-stage detectors. For a broader explanation in several deep learning concepts used in the detection context, be referred to (Jiao et al., 2019; Zhao et al., 2019).

**Two-stage detectors:** Two stage detectors are composed of a Region Proposal Network (RPN) and a Region of Interest Pooling (RoI-Pool) (Du et al., 2020) and have demonstrated high accuracy values in well-known datasets, such as MS COCO (Lin et al., 2014) and PASCAL VOC (Hoiem et al., 2009). These detectors are also capable of performing enhanced detail extraction even in small-size regions (Jana and Mohanta, 2022). The two-stage detectors work by first sending the images to a Convolutional Neural Network (CNN) backbone that extracts a feature map, similar to how human vision focuses on local salient details of images, and then, the RPN slides a window over the map to obtain fixed feature regions. Finally, the RoI-Pool layer samples the regions and reduces their dimensionality without a considerable loss of features and sends them t a SVM for classification and to a regressor for bounding box coordinate prediction (Li et al., 2017; Xie et al., 2021). The pioneering work of this approach, R-CNN (Girshick et al., 2014) employed a selective search algorithm for RPN, but it extracts the features from proposed image regions and not from proposed feature maps. Its enhanced version, fast R-CNN (Girshick, 2015), followed the aforementioned order since applying the RPN on the feature maps and later classifying them is faster thanks to compression. Later models, such as faster R-CNN (Ren et al., 2016) or mask R-CNN (He et al., 2017), aimed to improve the feature extraction section or the classification head, in terms of accuracy, context generalization, and timing. More recent methods had extended the original R-CNN framework to include components from Visual Transformers (ViT) as they have a current trend to held top positions in MS-COCO classification task. For instance, (Liang et al., 2022a), proposed an improved sparse R-CNN that exploit the sparsity in the region proposal and feed that information to attention units to focus on relevant global visual details rather than local ones such as with convolutional layers as they process the whole image at once. They proved the benefits of the method by testing for traffic sign detection. On the different manner, (Li et al., 2022), replace the ResNet50 backbone with a transformer version of EfficientNet known as EfficientFormer that could account for +3.9 AP score while alleviates intensive hardware usage. Some applications for object detection that look to accomplish robust prediction in real traffic scenarios took another path rather than extending backbones or adding slight changes to R-CNN. (Du et al., 2022). introduced an unknown-aware hierarchical object detection which incorporates *a priori* knowledge to distinguish between known classes and unknown classes that could be possible part of a higher taxonomy such as bicycles is a two-wheeled vehicle and vehicle itself is a class. Lastly, a hybrid detector was presented by (Khan et al., 2023) where the YOLO detection head was plugged with the results to RoI pooling stage from a faster R-CNN, thus eliminating the ROI proposals and reducing the computation overhead considerably while improving faster R-CNN results.

**One-stage detectors:** Two stage detectors give enhanced object detection, however, it slows down the overall detection process considerably (Carranza-García et al., 2020). Thus, an alternative approach, which achieves reasonably good detection with enhanced computational efficiency for real-time applications and safety-critical motion planning and decision making tasks, needed to be explored specifically for autonomous driving. As a viable alternative, one-stage detectors have emerged which work by introducing a single end-to-end (all layers are trained together) CNN to predict the bounding box class and coordinates. One of the pioneers of this strategy was You Only Look Once (YOLO) (Redmon et al., 2016) which divides the image into a grid and obtains the bounding box from each cell through regression. At the initial phase, the bounding boxes are anchor boxes with predefined sizes and they are used to tile the whole image. Depending on the results of class probability and Intersection-of-Union (IOU) scores, with respect to the ground truth annotation, the regressor refines the anchor boxes through manipulation of their center offsets. Although YOLO paved the pathway for numerous novel approaches in the domain of one-stage detectors, its major shortcoming is that it suffers localization accuracy for small objects, in comparison to two-stage detectors. Thus YOLO was further modified to reduce the accuracy gap between the two-stage and one-stage methods. The latest version, YOLOv8 (Jocher et al., 2023), has a pyramidal feature backbone for multi-scale detection and does not use anchor boxes but directly predicts the center of the bounding box. The method then applies mosaic augmentation to improve the training performance. Some of the other relevant one stage detectors which have been used in practce include, DCNv2 (Wang et al., 2020), which uses deformable CNN (DCN) to adapt to different geometric variations not contemplated by the fixed square kernel common in convolutions, and RetinaNet (Wang et al., 2020), which proposed a robust loss function to address false negatives due to the imbalance in the dataset between background and labeled classes. (Lyu et al., 2022). introduced RTMDet as a revision of YOLO detector with extensive modifications in taxonomy to account for real time inference, i. e., replacing convolutions with large-kernel depth-wise convolutions followed by point wise. The model achieves both increase in speed and mAP score. The ViT detectors have also took notoriety for one-stage detectors specially as these networks usually do not include RoI proposals to rather just have an accurate end-to-end prediction, though they tend to be slower than one-stage convolutional approaches. (Liu et al., 2021a). is capable of extracting features at various scales due to a shifted window scheme that simultaneously limits the self-attention computation which grows exponentially when the network gets deeper. (Ding et al., 2022). enhances SwinViT by introducing dual attention units that process spatial and channel tokens for a better understanding of global and local context respectively. Regarding autonomous driving applications, (Liang et al., 2022b), proposed incorporating attention units in a new lightweight backbone called GhostNet to considerable reduce mode size and increase inference speed. They tested the method along several augmentation techniques aim to have a more robust traffic sign detection under light condition changes. In a similar manner, DetecFormer (Liang et al., 2022c) was introduced by fusing local and global information in a global context encoder with the same purpose of traffic scene detection.

## 3 Datasets and evaluation metrics

With the advent of autonomous navigation in highly dynamic urban environments and under various weather conditions for ADS and connected autonomous driving, the intelligent transportation and machine vision research communities have continuously cultivated large datasets in the context of object detection for autonomous vehicles. The rapid takeoff of these datasets has been a major factor for the emergence of deep learning methods. This section summarizes 8 publicly accessible datasets for 3D object detection: KITTI (Geiger et al., 2013), nuScenes (Caesar et al., 2020), Waymo Open (Sun et al., 2020), Canadian Adverse Driving Conditions (CADC) (Pitropov et al., 2020), Boreas (Burnett et al., 2023), DAIR-V2X (Yu et al., 2022), A9 (Zimmer et al., 2023), ROPE 3D (Ye et al., 2022) datasets.

**Scene coverage:** The KITTI dataset provides 50 scenes captured in Karlsruhe, Germany across 8 classes in which vehicles, pedestrians, and cyclists are taken into account for online evaluation out of the 8 classes. The height of the 2D bounding boxes, the level of occlusion, and the degree of truncation are factors that are taken into account in determining the 3 difficulty categories, namely, easy, moderate, and hard. While, nuScenes captures 1*k* sequences from Boston and Singapore across 23 classes, only 10 classes are considered for evaluation. In addition, Waymo Open contains 1,150 sequences with 4 classes captured in Phoenix and San Francisco and similar to KITTI, there are 3 testing categories. As a pioneer, KITTI has had a significant impact establishing the standard for data collecting, protocol, and benchmark. The nuScenes and Waymo Open datasets both collect data throughout the day in a variety of weather and illumination conditions. Frequently, class imbalance is a problem that affects real data collection. As reported in (Qian et al., 2022), for the nuScenes and KITTI datasets, 50% of the categories account for 6.9% of the annotations indicating a long tail distribution.

Both CADC and Boreas are highly focused on adverse driving conditions and cover a wider spectrum of harsh weather conditions in comparison to the aforementioned datasets. The CADC dataset tracks 12.94 km of driving along 75 driving scenarios over 3 days in the Canadian Waterloo region during March 2018 and February 2019. A key difference in the CADC dataset is that their images were captured in numerous winter weather conditions and specific perceptually-degraded circumstances. Each sequence were given under different snowfall levels (e.g., light, medium, and extreme). The driving sequences were recorded with 8 cameras, 4 facing forward and 4 backward. The Boreas dataset involves 350 km of driving over 44 sequences in Toronto, Canada between 2020 and 2021 recorded in 2 repeated routes. The weather conditions change from day to night, snow to rain, and cloudy scenes. The dataset has a diversity of seasonal-variations over an extended period of time to further generalize the learning process. Thus, it seeks to provide opportunities for robust 3D detection under long-term weather changes for the same scenarios in highly dynamic urban driving conditions. We make a special distinction for V2X (Vehicle to everything) or V2I (Vehicle to infrastructure) datasets which provide multiple road views, i. e., front car and an elevated view due to the fact that visual multi-modality tackle occlusion and enhance detection robustness. One of the pioneers on this kind is DAIR-V2X which covers 10 km of city roads, 10 km of highway, 28 intersections, and 38 km^2^ of driving regions with diverse weather and lighting variations from the camera view and a pole elevated view. Each scene was recorded for 20 s to capture dense traffic flow and the driving of the experiment car on a unique intersection. In contrast, A9 provides higher scenery complexity as it contemplates various driving maneuvers, such as left and right turns, overtaking, and U-turns in different road locations throughout Munich, Germany; though only from infrastructure view. ROPE3D went even further achieving a broader generalization by collecting data in different weather conditions, illuminations and traffic density. In addition, authors took consideration on having spread distribution over annotations and depth of coarse-categories.

**Dataset size:** The KITTI dataset is one of the most popular datasets for use in autonomous driving and navigation. It contains 200*k* boxes which are manually annotated in 15*k* frames. Among this, it has 7,481 samples for training and 7,518 for testing, respectively. The training data has been further split into 3,712 samples for training and 3,769 for validation. In addition, the 1.4*M* labelled boxes in the nuScenes dataset are from 40*k* frames with 28,130, 6,019, and 6,008 frames used for training, validation, and testing respectively. Moreover, 112*M* boxes are annotated in Waymo Open dataset from 200*k* frames with 122,200, 30,407, and 40,077 for training, validation, and testing respectively. These datasets does not include annotations for testing and are rather internally evaluated. The CADC dataset consists of 7,000 instances expanded to 56,000 images from 8 cameras. Its 308,079 labels have been divided in 10 classes, including cars (281,941 labels), trucks (20,411 labels), buses (4,867 labels), bicycles (785 labels) and horses and buggies (75 label). Each class has a set of attributes which gives further semantic details, for instance transit bus for class of bus. The Boreas dataset consists of 37 training scenes and 16 test scenes that in conjunction contain 326,180 3D box annotations. The dataset contains vehicles, cyclists, pedestrians and miscellaneous (transit buses, trucks, streetcars, and trains). DAIR-V2X contains 71,254 camera frames with 40% and 60% from infrastructure and vehicle respectively and covers 10 classes including pedestrians, cars, buses and cyclists. A9 dataset collected 4.8 k images with 57.4 k manually labeled 3D boxes purely from infrastructure view. As other datasets, it covers 10 classes for classification task. At last, ROPE 3D holds 50 k images with the huge increment of 1.5 M 3D annotations over the last two datasets. It also has a slight increase in classification difficult as it includes 13 classes with labels such as *unknown-unmovable*, *unknown-movable* or *traffic cone*.

**Evaluation metrics:** Similar to 2D object detection, 3D object detection methods employ Average Precision AP as standard benchmark. The standard AP metric is first established, followed by the AP variants that have adopted considerations for a series of predictions $\left\{y_{1},\ldots ,y_{n}\right\}$ that are listed in decreasing order of confidence score *s*_*i*_. A prediction **y**_*i*_ (bounding box and class) is regarded as a true positive if the ratio of the intersection of the are covered by the prediction bounding box *B* and its ground truth correspondence, known as the Intersection over Union (IoU), exceeds a predetermined threshold; otherwise, it is regarded as a false positive. The AP score is defined as the area of the region beneath the precision-recall curve, which graphically resembles a zigzag pattern. Since it is challenging to determine the area under the curve numerically, Interpolated ${\left.AP\right|}_{R_{N}}$ was introduced by PASCAL VOC (Everingham et al., 2010) as a numerical approximation. It is formulated as the mean precision calculated for *N* levels from a recall subset *R*, given as

$${\left.AP\right|}_{R_{N}}=\frac{1}{N}\underset{r\in R}{\sum }P_{\text{interpolate }}\left(r\right)$$ (1)

where *r* takes values from a evenly-spaced set of N numbers [0, 0.1, 0.2, … 1] which follows a decreasing trend in P-R curve. Consider that the interpolated function of *P*(*r*) must be evaluated such as the precision for recall *r* is the maximum value for all recall values *r*′ greater than the reference *r* recall. It should be mentioned that the CADC dataset has not published its 3D detection benchmark yet.

### 3.1 KITTI benchmark

The metric used for detection benchmarking in KITTI is the interpolated AP_11_ metric. The considered scores in leaderboard ranking are test AP from bird eye view (BEV) detection and 3D detection. The evaluation for car, pedestrian, and cyclist accounts for different IoU thresholds in AP calculation. The passenger vehicle class uses 0.7 and the others 0.5 because of the occlusion frequency of each class. Changes were applied to the amount of recall levels, from 11 levels [0, 1/10, 2/10, … , 1] to 40 levels [1/40, 2/40, 3/40, … , 1] with recall level 0 being removed as proposed by (Simonelli et al., 2019b).

### 3.2 nuScenes benchmark

The official evaluation statistic for nuScenes is the nuScenes Detection Score (NDS), which is a set of mean average errors in translation, size, orientation, attribute, and velocity given by:

$$\text{NDS}=\frac{1}{10}\left[5\text{mAP}+\underset{\text{mTP}\in \text{TP}}{\sum }\left(1-\min\left(1,\text{mTP}\right)\right)\right],$$ (2)

where, mAP indicates mean Average Precision and TP is the set of the 5 mean true positive metrics calculated for each class. The mAP is calculated over *C* classes and *D* distance thresholds of values [0.5,1,2,4] meters. While obtaining the AP and before computing means, any operational point with precision or recall less than 10% is discarded.

### 3.3 Boreas benchmark

This dataset follows the KITTI dataset scheme. For a passenger vehicle, a 70% overlap threshold in mAP calculation count is considered as true positive and 50% for pedestrians.

### 3.4 Waymo benchmark

The Waymo Open dataset proposes a heading version of AP called APH which incorporates a heading function with respect to the recall similar to the calculation of area under the curve of the Precision/Recall (PR) plot. Each true positive is weighted by heading accuracy defined between $\min\left(\left|\theta -\theta^{*}\right|,2\pi -\left|\theta -\theta^{*}\right|\right)/\pi$ . Here, *θ* and θ* indicate the predicted azimuth angle and the corresponding ground truth, within [−*π*, *π*]. Similar to AP, APH is normalized in range of [0,1]. To obtain the recall gap, Hungarian matching is performed for the prediction above a specified threshold. A threshold of 0.7 for vehicles and 0.5 for pedestrians is used. This matching is used for calculation of precision and recall. If in the APH calculation, the recall gap is above the default value of 0.05, more operation points are added to avoid over-estimation.

### 3.5 DAIR-V2X benchmark

It employs the same AP score as (Everingham et al., 2010) and introduces the transmission cost as the average send bytes between infrastructure and vehicle. In V2X detection it is common to have two separate detectors that interact which each other at different stages, may be early or late fusion. Since there is physical separation between the two data stations, it is highly relevant to minimize the data transmission between the stations. Nonetheless, there is a trade-off between efficient transmission and lost information so it is valuable to compare AP score and transmission cost together across different detectors.

### 3.6 A9 benchmark

It does not provide an official metric for 3D detection task.

### 3.7 ROPE 3D benchmark

It not only adopts the AP_40_ variant from (Simonelli et al., 2019c), but also computes similarity scores for ground center (ACS), orientation (AOS), ground occupancy area (AAS), four ground point distance (AGS) and fuses them. Assume S = (ACS + AOS + AAS + AGS)/4, their fuse score known as ROPE_*score*_ is equal to:

$${\text{ROPE}}_{\mathrm{score}}=\frac{\omega_{1}AP+\omega_{2}S}{\omega_{1}+\omega_{2}}$$

where the weights *ω*_1_ = 8 and *ω*_2_ = 2, thus giving a higher importance to AP detection metric.

Please, refer to (Ye et al., 2022) for greater detail on those.

## 4 Three-dimensional object detection

**FIGURE 2:**
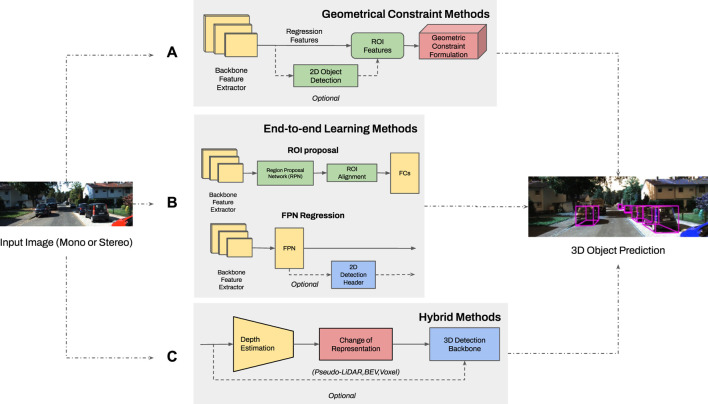
The structure of existing 3D object detection methodologies (having the same input of monocular or stereo images and output of the 3D detection header): **(A)** Methods using geometrical constraints use ROI features from backbone output or combine them with 2D bounding boxes to fit constraints on loss function or space projection. **(B)** End-to-end learning methods update all layer parameters using backpropagation. This method is categorized depending on utilization of an ROI or feature pyramid network regression with an optimal 2D detection. **(C)** Hybrid methods combine depth estimation from a standalone pretrained network and a change of representation to leverage detailed features for 3D detection. The 3D backbone can be from existing methods for LiDAR, BEV or Voxel points.

The 3D object detection problem has an added level of complexity as compared to 2D detection, since it localizes the objects with respect to the camera and identifies the orientation/heading through fitting of 3D bounding boxes. The detector input can be monocular or stereo data, where each kind of detector leverages its input in different ways. An overview of 3D object detection methodologies is shown in Figure 2 where the classification is in three main categories: model based methods (which leverage geometrical constraints applicable in autonomous driving context), end-to-end learning, and hybrid methods.

**FIGURE 3:**
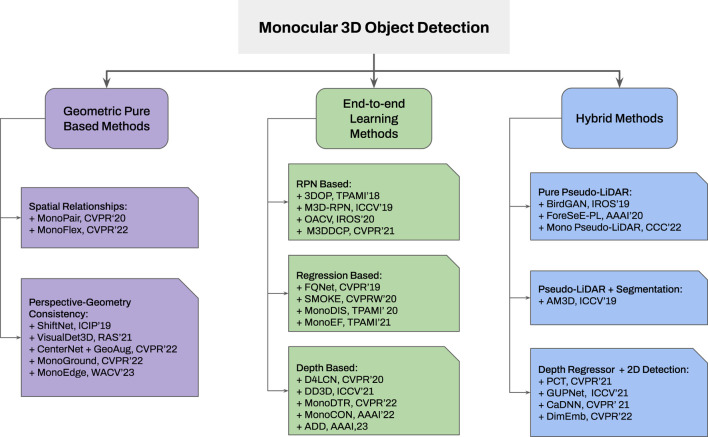
Taxonomy of monocular 3D object detection frameworks: *i*) Geometric methods consider spatial relationships between several objects and perspective consistency; *ii*) The end-to-end learning framework is categorized based on their utilization of internal features; and *iii*) Hybrid methods were classified by 3D representation and its augmentation with other techniques such as segmentation or 2D detection.

**FIGURE 4:**
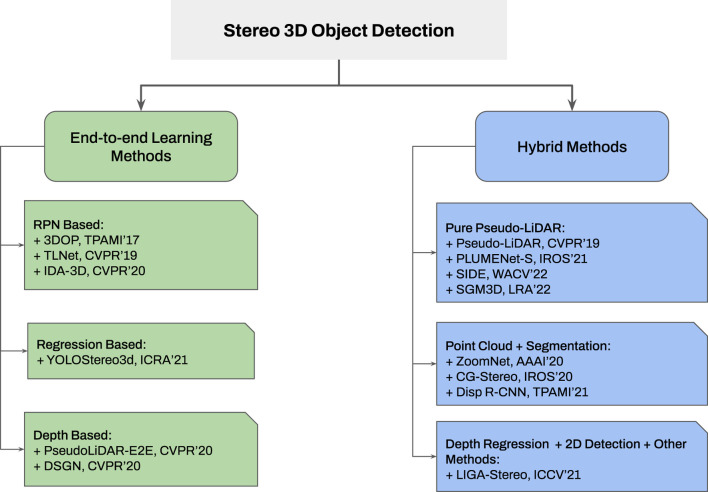
Taxonomy of stereo 3D object detection approaches. None-geometrical methods are widely utilized for stereo vision based 3D object detection since previously trained depth estimators or end-to-end depth cost volume achieve better results compared with geometric methods (utilizing in stereo camera). For the remaining categories, the inner classification remains the same as monocular 3D object detection frameworks.

Additionally, two classification diagrams are provided in Figures 3, 4 for monocular and stereo visions, respectively.

### 4.1 Model based approaches

This subsection discusses model based approaches which mainly use monocular 3D detectors and geometric constraints in street view to make accurate depth estimation or directly utilize box regression. Stereo detectors mostly relay information through end-to-end learning depth networks or more complex 3D representations instead of leveraging geometric information. The constraints are formulated explicitly in a custom layer or loss function, i.e., epipolar or projection model constraints. Also, they can be integrated in the form of geometric projections or transformations. To feed the geometric formulation stage, the detector backbone passes its features from a set of ROIs or fuse those with 2D object detection predictions, as shown in the first block of Figure 2. Its output is delivered to a detection header for the 3D box prediction. The numerical results regarding computational speed and AP for different classes and categories are reported in Table 1 for a detailed comparison. In (Roddick et al., 2018), the authors proposed OFT-Net (Orthographic feature transformation), which is a network that projects multi-scale features from a ResNet-18 backbone into a 3D orthographic space using the camera’s intrinsic parameters. The new projection gives a better representation of the 3D space than the pinhole projection since it is robust to appearance and scale distortions due to poor depth inference. Besides, the rest of the pipeline follows the standard classification task by bounding box regression and NMS. The authors of (Naiden et al., 2019) extended the Faster R-CNN head to predict 3D bounding box dimensions and angles apart from the 2D detection task. A least-square problem is formulated by 3D geometric constraints. The system of equations considers projection matrix constraints and enforces the 3D box edges to fit inside 2D box sides. A closed-form solution to this problem is given in (Naiden et al., 2019) where the box translation vectors are determined. The 3D parameters and 2D initial estimation are then passed to a ShifNet network for refinement through a newly proposed Volume Displacement Loss (VDL) that aims to find the translation which optimizes IoU between two 3D predicted boxes while fixing depth and angle.A 3D anchor preprocessing scheme and a custom layer called Ground-Aware Convolution (GAC) module is proposed in (Liu et al., 2021b) that infers depth via perspective projection from the camera to the ground since ground-level is a metric reference and is known *a priori* by car dimensions. Their basis is that ground-awareness gives enough clues for depth estimation and leverages it to do 3D detection. The preprocessing stage backpropagates the anchors to 3D space and rejects those far from ground-level. Afterward, the filtering results feed the GAC module to estimate vertical offsets from the ground and then compute depth priors with perspective geometry. Its output is a depth feature map included in a 3D regression head. In (Lian et al., 2022a) the effect of 4 geometric manipulation augmentations in 3D detection training has been explored. They studied the inaccuracy of depth estimation under different object positions and sizes. In order to make 3D detectors more robust to these geometrical distortions, they proposed random cropping, random scale, moving camera, and copy-paste augmentations. The first 2 methods are widely popular for 2D detection community. The third technique utilizes depth to translate the instance pixels to the augmented image. The copy-paste processing samples an object, takes it out of its context and pastes it into another region. It ensures geometrical consistency for the object in terms of position and angle concerning the background scene. Including these augmentations in the training stage, enhanced the accuracy of previous detectors. Further, local perspective distortion on 3D objects to infer depth and global yaw angle without using camera parameters has been explored in (Zhu et al., 2023). They introduced the concept of keyedges and keyedges-ratio to parameterize 3D bounding box vertical edges locally. The keyedges-ratio is used directly in regressing depth and yaw. From each box, they get 4 keyedges and subsequently 4 depth predictions which they fuse using an uncertainty-based operation. Subsequently, the local-perspective results are merged with global perspective effects in other monocular 3D detectors such as MonoFlex (Gu et al., 2022). Hence, both image perspectives enrich the extracted visual content for detection.

**TABLE 1:** Geometrical constrained model based methods comparison table. Best results are highlighted in **bold** font. The AP scores for car category were calculated considering IOU (Intersection of Union) of 70%, as required for submission to KITTI oficial evaluation.

Method	Source	FPS	Camera input	KITTI dataset validation set (AP_3*D*_/AP_*BEV*_)		
				Cars		
				Easy	Moderate	Hard
OFT-Net	Roddick et al. (2018)	-	Mono	4.07/11.06	3.27/8.79	3.29/8.91
GS3D	Li et al. (2019b)	0.23	Mono	13.46/-	10.97/-	10.38/-
MonoPair	Chen et al. (2020a)	17.54	Mono	16.28/24.12	12.30/18.17	10.42/15.76
ShiftNet	Naiden et al. (2019)	3.86	Mono	13.84/18.61	11.29/14.71	11.08/13.57
VisualDet3D	Liu et al. (2021b)	20	Mono	23.63/-	16.16/-	12.06/-
MonoFlex	Gu et al. (2022)	33.33	Mono	21.75/29.60	14.94/20.68	13.07/17.81
CenterNet + GeoAug	Lian et al. (2022a)	33.3	Mono	24.53/-	17.23/-	14.32/-
MoNet3D	Zhou et al. (2020)	27.85	Mono	22.73/27.48	16.73/21.80	15.55/17.86
MonoGround	Qin and Li (2022)	33.3	Mono	25.24/32.68	18.69/24.79	15.58/20.56
MonoEdge	Zhu et al. (2023a)	-	Mono	**25.66/33.71**	**18.89/25.35**	**16.10/22.18**

### 4.2 End-to-end learning based methods

End-to-end learning refers to the capability of updating all the parameters in a network with a single loss function such that backpropagation takes place from the head up to the network backbone. In consequence, the learned representations of depth, geometry, or 3D space overall gets embedded in all network layers and this results in minimization of time and enhanced prediction accuracy. A comparison between the state-of-the-art end-to-end learning methods is presented in Table 2. The work in (Bao et al., 2020) falls under a category where an end-to-end trainable monocular detector is designed to work effectively even without learning dense depth maps. The working principle behind this method involves projecting the grid coordinates from the 2D box to 3D space followed by developing an object-aware voting model. Such voting models use appearance attention and distribution of geometric projection to find proposals for the 3D centroid, thereby, facilitating object localization. Another end-to-end approach in (Liu et al., 2020), predicts the 3D bounding boxes by combining single key-point estimates and regressed 3D variables. The advantage of this method is that it also works on a multi-step disentangling approach resulting in improved convergence of training and detection accuracy. In (Zhou et al., 2021), the camera pose is captured to propose a detector free from extrinsic perturbation. This framework is capable of predicting the extrinsic parameters of the camera through effective detection of change in the horizon as well as through the use of vanishing point. Further, a converter is designed to enable the 3D detector to work independent of any extrinsic parameter variations.

**TABLE 2:** End-to-end learning methods comparison. Best results are highlighted in **bold** font. The superscript ^⋆^ in results correspond to test set scores (since only those scores were available). The AP scores for car category were calculated considering IOU (Intersection of Union) of 70% while for pedestrians and cyclists categories was 50%, as required for submission to KITTI oficial evaluation.

Method	Source	FPS	Camera input	KITTI dataset validation set (AP_3*D*_/AP_*BEV*_)								
				Cars			Pedestrians			Cyclists		
				Easy	Moderate	Hard	Easy	Moderate	Hard	Easy	Moderate	Hard
FQNet	Liu et al. (2019)	-	Monocular	5.98/-	5.50/-	4.75/-	-	-	–	-	-	-
MLF-3D	Xu and Chen (2018)	-	Monocular	10.53/-	5.69/-	5.39/-	-	-	-	-	-	-
DST3D	Wu et al. (2022)	12.5	Monocular	13.46/17.33	11.28/14.83	11.06/14.18	-	-	-	-	-	-
OACV	Bao et al. (2020)	-	Monocular	13.65/20.65	11.47/16.35	10.70/14.21	11.5/13.10	10.93/12.33	10.04/11.70	-	-	-
SMOKE	Liu et al. (2020)	33.33	Monocular	14.76/19.99	12.85/15.61	11.50/15.28	-	-	–	-	-	-
MonoEF	Zhou et al. (2021)	33.3	Monocular	21.29/29.03^⋆^	13.87/19.7^⋆^	11.71/17.26	-	-	-	-	-	-
MonoRUn	Chen et al. (2021b)	14.29	Monocular	20.02/-	14.65/-	12.61/-	-	-	-	-	-	-
MonoDIS		-	Monocular	18.05/24.26	14.98/18.43	13.42/16.95	10.79/11.04	10.39/10.94	9.22/10.59	5.27/5.52	4.55/4.66	4.55/4.55
TLNet	Qin et al. (2019)	-	Stereo	18.15/29.22	14.26/21.88	13.72/18.83	-	-	-	-	-	-
OPA-3D	Su et al. (2023)	25	Monocular	19.40/25.51	24.97/33.80	16.59/22.13	-	-	-	-	-	-
MonoDETR + ADD	Zhang et al. (2023)	-	Monocular	25.30/34.14	16.64/23.49	14.90/21.24	-	-	-	-	-	-
M3D-RPN	Brazil and Liu (2019)	6.21	Monocular	20.27/25.94	17.06/21.18	15.21/17.90	-	11.28/11.60	–	-	10.01/10.13	-
MonoDTR	Huang et al. (2022)	27	Monocular	24.52/33.33	18.57/25.35	15.51/21.68	-	-	-	-	-	-
M3DSSD	Luo et al. (2021)	-	Monocular	26.95/44.42	18.68/29.69	15.82/24.60						
MonoCon		38.7	Monocular	26.33/34.65	19.01/25.39	15.98/21.93	-	-	-	-	-	-
D4LCN	Ding et al. (2020)	-	Monocular	26.97/-	21.71/-	18.22/-	-	-	-	-	-	-
DD3D	Park et al. (2021)	-	Monocular	23.22/30.98^⋆^	16.34/22.56^⋆^	20.03/14.20^⋆^	13.91/15.90^⋆^	9.30/10.85^⋆^	8.05/8.05^⋆^	2.39/3.20^⋆^	1.52/1.99^⋆^	1.31/1.79^⋆^
ProGen-Net	ul Haq et al. (2022)	7.63	Monocular	31.3/37.1	25.9/32.7	20.7/23.5	-	-	-	-	-	-
YOLOStereo3D	Liu et al. (2021c)	12.5	Stereo	65.68/-	41.25/-	30.42/-	28.49/-	19.75/-	16.48/-	-	-	-
Stereo RCNN	Li et al. (2019a)	3.57	Stereo	54.11/68.50	36.69/48.30	31.07/41.47	-	-	-	-	-	-
IDA-3D	Peng et al. (2020)	-	Stereo	54.97/70.68	37.45/50.21	32.23/42.93^⋆^	-	-	-	-	-	-
PseudoLiDAR-E2E	Qian et al. (2020)	2.04	Stereo	71.1/82.7	51.7/65.7	46.7/58.4	**32.3/35.7**	**24.9/27.8**	**21.5/23.4**	**38.4/42.8**	**24.1/26.2**	**22.7/24.5**
DSGN	Chen et al. (2020b)	1.47	Stereo	72.32/83.24	54.27/63.91	47.71/57.83	-	-	-	-	-	-
3DOP	Chen et al. (2017)	0.83	Stereo	**90.43/-**	**68.90/-**	**62.22/-**	-	-	-	-	-	-

For a well-known end-to-end framework called MonoRUn, an uncertainty-aware reconstruction network is designed in order to regress the pixel-related 3D object coordinates, and for the training, the predicted 3D coordinates are projected back on to an image plane in (Chen et al., 2021). An approach that uses a disentangling transformation for losses in detection, along with generating a confidence score based on self-supervised learning is proposed in (Simonelli et al., 2019) which do not need class labels. While (Zhang et al., 2023) introduces a framework that transforms into a depth-aware detection process and represents 3D object candidates through set queries. Then, an attention encoder based on depth is utilized to produce a non-local depth embedding from the image which was provided as input. Further, a depth-guided decoder is then used for inter-query and query-scene depth feature interactions leading to adaptive estimates of each object query. Leveraging the geometric relationship between the 2D and 3D outlook while enabling 3D boxes to use convolutional features produced in image-space; an object detection algorithm is proposed in (Brazil and Liu, 2019). Depth-aware convolutional layers are also designed in this work which enables location-specific feature development, in turn improving the understanding of 3D scenes.

An end-to-end depth-aware transformer network for 3D object identification in monocular vision consisting of feature enhancement and transformer model is proposed in (Huang et al., 2022). While the authors of (Luo et al., 2021), introduce an approach where first a shape alignment is carried out followed by the center alignment. This combined with an attention block to extract depth features improves the overall performance of the proposed algorithm. Learned auxiliary monocular contexts are utilized in (Liu et al., 2022), which uses 3 components, namely, a feature backbone based on Deep Neural Network (DNN), learning parameters using regression head branches, and learning auxiliary contexts using regression head branches. A single-stage detector that benefits from pre-training of depth, and with efficient transfer of information between the estimated depth and detection, while allowing scaling of the unlabeled pre-training data is proposed in (Park et al., 2021). Regressing the dimensions along with the orientation through the use of an anchor-based approach, such that a 3D proposal can be constructed is introduced in (Liu et al., 2019).

It is not only through the use of monocular cameras that end-to-end approaches have been found to provide efficient 3D object detection but with stereo cameras as well. In (Qin et al., 2019), 3D anchors are employed to design correspondences at the object level, in between stereo images. This enables DNNs to effectively learn and in turn detect the object of interest in 3D space. Incorporating inference structure as well as knowledge gathered in real-time, a 1-stage detector is proposed with a stereo matching module which is lightweight as discussed in (Liu et al., 2021c). A method, called Stereo R-CNN (Li et al., 2019), is known to associate and detect objects in either side of images simultaneously. Extra branches are also added in order to predict the dimensions of objects and sparse key-points. These are then combined with the 2D left-right boxes for obtaining a coarse 3D object bounding box. Finally, the accurate 3D bounding box is recovered through a region-based photometric alignment using left and right RoIs. In (Peng et al., 2020), only RGB images are taken and 3D bounding boxes are annotated as the training data. As an all important factor, the depth estimations are considered and an Instance-Depth Aware module is introduced to predict the depth of the centre of the bounding box. A framework which is based on the differentiable Change of Representation modules and which trains the entire PL pipeline end-to-end is proposed in (Qian et al., 2020). Based on how representations of 3D scenario prediction is to take place, a method called Deep Stereo Geometry Network was proposed in (Chen et al., 2020b). This approach detects 3D objects on a differentiable volumetric representation thus encoding 3D geometric structure for 3D regular space. Another methodology of object detection that works by minimizing an energy function and encodes priors of object sizes, defines object placement on the ground plane in addition to several depth informed features while utilizing CNN is discussed in (Chen et al., 2017). We make an special mention to the multi-view end-to-end object detectors because they showed a significant boost in robustness in terms of adversarial attacks and poor depth representations (Xie et al., 2023; Zhu et al., 2023; Jiang et al., 2023) states that the polar coordinates suits as a more natural 3D world representation in bird’s eye view thus they proposed a cross attention based Polar detection head where they re-parametrized projection models and grid structure to use polar coordinates. As input, the model uses 6 cameras views that sweep the car’s polar view. (Li et al., 2023a). studied extensively the deficiencies inside depth modules in current multi-view 3D object detector and introduced BEVDepth which is trained with supervision module from LiDAR point cloud to apply corrections in the predicted depth distribution of each view. This accomplishes more accurate depth predictions, avoids depth overfitting and helps to obtain better BEV semantics inference. In (Li et al., 2023b), the authors rejected ViT based detectors due to their internal quadratic operations, i. e., cross attention. They rather proposed a fully convolutional multi-view detector which reports similar AP as (Li et al., 2023a) but with an increase of 3 times in inference speed. They managed to implemented pure convolutional depth estimation, fusion module and BEV encoder thus obtaining a linear computational cost. (Xiong et al., 2023). explored prioritizing local feature in camera view rather than global ones because using them for learning view transformation was trickier due to inaccuracies in extrinsic parameters. Their network called CAPE employed feature-guided key position embedding for local features and a query position encoder for global ones to later fuse both in a single encoder.

### 4.3 Hybrid approaches

Before end-to-end learning methods could reach performance comparable to LiDAR detectors, exploiting new 3D representations such as Pseudo-LiDAR or BEV were proposed in literature and applied in practice to have finer feature extraction and reduce the performance gap. Moreover, the detection head was also inspired in LiDAR 3D detection or other frameworks and the list of these methods are summarized in Table 3. In that sense, hybrid methods aim to exploit previously proposed methods, from model-based or end-to-end learning methods, as the depth estimators and then introduce an internal change of representation to exploit 3D detectors originally designed for other frameworks such as LiDAR detection. As the depth network were already trained and achieved a reasonable performance, researchers only need to focus in defining the appropriate representation and its conversion for detection. One such hybrid approach is proposed in (Wang et al., 2021) where the authors have utilized a lightweight strategy for obtaining learned coordinate representations. An approach by which the localization can be enhanced and which introduces confidence-aware loss is used for prediction. Such hybrid approaches have been found to be up to the task of effectively and efficiently tackling the problem of localization in literature and in practice. Using a similar hybrid approach in (Reading et al., 2021), the predicted depth distribution is used in order to project the feature information in 3D space. Then through the use of bird’s-eye-view projection with a single-stage detector, the output detection is obtained. In another approach (Wang et al., 2020) the data distribution is analysed continued by a scan of the interactions in the background and foreground, followed by a separated depth estimation based on ForeSeE method for estimating their respective depths. While in (Zhang et al., 2022b) using DNNs a pair-wise distance is exploited for obtaining the similarity of dimensions so that the proposed model has the option of exploiting the inter-object information to learn further for more effective dimension estimation.

**TABLE 3:** Hybrid methods comparison table. Best results are highlighted in **bold** font. The superscript ^⋆^ in results correspond to test set scores (since only those scores were available). The AP scores for car category were calculated considering IoU (Intersection of Union) of 70% while for pedestrians and cyclists categories was 50%, as required for submission to KITTI oficial evaluation.

Method	Source	FPS	Camera input	KITTI dataset validation set (AP_3*D*_/AP_*BEV*_)								
				Cars			Pedestrians			Cyclists		
				Easy	Moderate	Hard	Easy	Moderate	Hard	Easy	Moderate	Hard
PCT	Wang et al. (2021a)	22	Monocular	13.37/19.03	21.00/29.65	11.31/15.92	-	-	-	-	-	-
CaDNN	Reading et al. (2021)	-	Monocular	19.17/-^⋆^	13.41/-^⋆^	11.46/-^⋆^	12.87/-^⋆^	8.14/-^⋆^	6.76/-^⋆^	7/-^⋆^	3.41/-^⋆^	3.3/-^⋆^
ForeSeE-PL	Wang et al. (2020b)	-	Monocular	15.0/23.4	12.5/17.4	12.0/15.9	-	-	-	-	-	-
GUPNet + DimEmb	Zhang et al. (2022b)	32.15	Monocular	23.62/32.82^⋆^	16.10/21.98^⋆^	13.41/18.70^⋆^	-	-	-	-	-	-
MonoJSG	Lian et al. (2022b)	23.81	Monocular	24.69/32.59^⋆^	16.14/21.26^⋆^	13.64/18.18^⋆^	11.02/-^⋆^	7.49/-^⋆^	6.41/-^⋆^	5.45/-^⋆^	3.21/-^⋆^	2.57/-^⋆^
GUPNet	Lu et al. (2021)	29.4	Monocular	22.76/31.07	16.64/22.94	13.72/19.75	-	-	-	-	-	-
SGM3D	Zhou et al. (2022)	33	Stereo/Monocular	25.96/34.10	17.81/23.62	15.11/20.49	-	-	-	-	-	-
FGMF-AC	Liu et al. (2022b)	6.25	Monocular	29.67/37.70	22.96/26.99	18.97/24.29	-	-	-	-	-	-
Pseudo-Mono	Tao et al. (2023)	-	Mono	27.41/35.84^⋆^	18.57/23.67^⋆^	16.16/20.19^⋆^	29.26/36.11	22.15/28.04	19.27/23.90	-	-	-
AM3D	Ma et al. (2019)	-	Monocular	32.23/-	21-09/-	17.26/-	-	-	-	-	-	-
Mono PseudoLiDAR	Wang et al. (2022b)	-	Monocular	32.4/42.5	21.4/29.1	17.3/24.7	-	-	-	-	-	-
PseudoLiDAR	Wang et al. (2019)	1	Stereo	59.4/72.8	39.8/51.8	33.5/44.0	33.8/41.3	27.4/34.9	24.0/30.1	41.3/47.6	25.2/29.9	24.9/27.0
SIDE	Peng et al. (2022)	3.85	Stereo	61.22/72.75	44.46/53.71	37.15/46.16	-	-	-	-	-	-
SAS3D	Gao et al. (2023)	35.71	Stereo	65.26/77.48	47.07/58.41	39.62/49.95	-	-	-	-	-	-
BirdGAN	Srivastava et al. (2019)	-	Monocular	58.26/-	42.48/-	40.72/-	-	-	-	-	-	-
PLUMENet-S	Wang et al. (2021b)	12.5	Stereo	-/74.4	-/61.7	-/55.8	-	-	-	-	-	-
ZoomNet	Xu et al. (2020)	-	Stereo	62.96/78.68	50.47/66.19	43.63/57.60	-	-	-	-	-	-
PseudoLiDAR + Geo	Li et al. (2022b)	28.57	Stereo	68.23/78.77	48.34/59.01	44.84/55.51	-	-	-	-	-	-
PseudoLiDAR++	You et al. (2019)	11.1	Stereo	67.9/82.0	50.1/64.0	45.3/57.3	53.6/63.7	44.4/53.8	38.1/46.8	60.8/65.7	40.8/45.8	38.0/42.8
CDN-DSGN	Chen et al. (2020b)	-	Stereo	74.5/83.3^⋆^	54.2/66.2^⋆^	46.4/57.7^⋆^	-	-	-	-	-	-
Disp R-CNN	Sun et al. (2020b)	2.59	Stereo	70.18/83.29	54.72/66.18	46.99/57.60	**43.87/50.70**	36.26/38.33	**29.81/33.50**	**55.98/61.60**	33.46/36.89	**29.51/35.07**
CG-Stereo	Li et al. (2020)	1.76	Stereo	76.17/87.31	57.82/68.69	54.63/65.80	-	-	-	-	-	-
LIGA-Stereo	Guo et al. (2021)	2.86	Stereo	**84.92/89.35**	67.06/77.26	**63.80/69.05**	-	-	-	-	-	-
DSGN++	Chen et al. (2022a)	5.62	Stereo		**69.12/78.93**		-	**42.44/50.06**	-	-	**42.48/45.77**	-

To benefit from the advantages of DNNs as well as the imposition of geometric constraints at the pixel level, the object depth estimation problem is re-formulated as a refinement problem in (Lian et al., 2022b). To reduce the feature degradation brought on by depth estimation errors, virtual image features are created using a disparity-wise dynamic convolution with dynamic kernels taken from the disparity feature map in (Chen et al., 2022). A separate module to convert the input data from a 2D plane to a 3D point cloud space for a better input representation is explored in (Ma et al., 2019). This is followed by the use of PointNet backbone net to conduct 3D detection to determine the positions, dimensions, and orientations of the objects in 3D space. A multi-modal feature fusion module to include the complementary RGB cues into the produced point cloud representation in order to improve the point cloud’s capacity to discriminate is also investigated in (Ma et al., 2019). Further, 2D object proposals are identified in the input image by using a pipeline of two-stage 3D detection methods, and a point cloud frustum from the pseudo-LiDAR for each proposal is extracted. Then each frustum’s oriented 3D bounding box is found and ways through which the noise in the pseudo-LiDAR can be dealt with are also discussed in (Weng and Kitani, 2019). Utilizing current networks that operate directly on 3D data to conduct 3D object recognition and localization while also employing neural networks to convert 2D images to 3D representations has been discussed in (Srivastava et al., 2019).

The use of stereo cameras with learning based techniques to obtain a hybrid approach has been discussed in detail in literature. One such approach (Wang et al., 2019) converts the image-based depth maps to pseudo-LiDAR representations, which are fundamentally imitative of the LiDAR signal, taking into account the inner workings of convolutional neural networks. Using this representation, several LiDAR-based detection techniques that are already available can be exploited. The stereo 3D detector is a stereo-image based anchor-free 3D detection approach in which the instance-level depth information is investigated in (Peng et al., 2022) by creating the cost volume from ROIs of each item. Due to the information scarcity of local cost volume, match reweighting is applied in addition to structure-aware attention to enhance the concentration of depth information. It suggests a shape-aware non-uniform sampling approach to make use of the pertinent data from the object’s exterior region. While utilizing trained neural networks to transform 2D images into 3D representations and using existing networks to operate directly on 3D data to produce better results is discussed in (Srivastava et al., 2019). A framework called ZoomNet is introduced in (Xu et al., 2020) for stereo imagery-based 3D detection that leverages a standard 2D item identification model and adaptive zooming to generate pairs of left-right bounding boxes. It also proposes the 3D fitting score to assess the 3D detection quality and the learning of component positions to increase resistance to occlusion.

A lightweight pseudo-LiDAR 3D detection system is proposed in (Li et al., 2022) that achieves responsiveness and accuracy by using Binary Neural Networks (BNNs) to increase the completeness of objects and their representation in 3D space. While a strategy in which a one-stage stereo-based 3D detection pipeline that simultaneously recognises 3D objects and calculates depth, closing the gap between semantic and depth information is discussed in (Chen et al., 2020b). Using a statistical shape model to produce dense disparity pseudo-ground-truth without LiDAR point clouds, broadening applicability and addressing the issue of lack of disparity annotation has been tackled in (Sun et al., 2020). To increase the efficiency of learning semantic features from indirect 3D supervision, a second 2D detection head was attached in (Guo et al., 2021), which enhanced the overall geometric and semantic representation. Also, depth-wise plane sweeping, dual-view stereo volume, and stereo-LiDAR Copy-Paste to lift 2D and 3D information to the stereo volume have been explored in (Chen et al., 2022). This is a multi-modal data editing technique to maintain cross-modal alignment and increase data effectiveness.

## 5 Trends in reliable three-dimentional object detection

From the presented results and analysis in this work, it can be projected that the object detection community is moving towards employing hybrid methods on stereo vision that leverage Pseudo-LiDAR representation and infer depth through dedicated networks or combine these approaches with geometric constraints. The current LiDAR based state estimation and detection approaches have provided a wide array of hybrid techniques and has also provided the background needed to propose new detectors without manipulating LiDAR data. Considering the best results in terms of AP_3*D*_/AP_*BEV*_ and its corresponding methods, they run at least in 30 FPS (real-time) and *SAS*3*D* (39.62/49.95) is the most prominent framework of the hybrid branch. It outperforms its counterparts of geometric, MonoGround (15.58/20.56), and end-to-end, MonoCon, (15.98/21.93) branches. Interestingly, end-to-end methods do not generally perform better than the model-based approaches in terms of inference time. This gives the impression that end-to-end approaches will still need further improvements in the case of real-time applications, and more specifically for safety-critical scenarios in highly dynamic settings. In particular, end-to-end methods that rely on a two-branch structure tend to be have higher AP but slower inference, similarly as with 2D detection. Conversely, model-based detectors obtain rapid results due to levering depth through geometric scene and not dense feature map, but this is also its weakest point since this process is more sensitive to depth artifacts. This branch can be use for real-time applications with sufficient awareness on AP results. Lastly, hybrid-methods, expressly those based on Pseudo-LiDAR representation, heavily depend on previous depth estimator regarding both in detection quality and speed, in other words it is its major bottleneck.

Several critical detection aspects are yet to be tackled which include, failure of CNN to capture the finest texture details while only focusing on local visual information and limitations in its extraction capabilities. As a possible remedy to such issues, visual transformers (ViT) have been proposed as the deep learning structure to obtain global visual information and to keep long-term spatial structures due to its embedding mechanism.

Several transformer-based frameworks, have already been presented in this survey, which include, MonoDTR (Huang et al., 2022), DST3D (Wu et al., 2022), and MonoDETr (Zhang et al., 2023). These frameworks have been used for precise depth inference or serves as feature backbones. A key feature of such methods is its end-to-end learning capabilities and its scalability. Also, it is concerning that only a few approaches have categorically addressed the occlusion problem, considering that most street scenarios suffer from varying degrees of occlusion. The KITTI dataset contemplates occlusion for mAP calculation by considering categories of Easy, Moderate, and Hard. As another possible solution to this issue, the authors of (Liu et al., 2022; Su et al., 2023) have exploited the anti-occlusion loss function which fuses depth and semantic information and defines a confidence occlusion parameter inside the loss. However, further investigation and analytical analysis must be carried out to effectively tackle the issue of occlusion.

An alternative approach of designing a part-aware mechanism to extract features from non-occluded parts of the vehicle, i.e., wheels or car plates was undertaken by ZoomNet (Xu et al., 2020). Those parts can guide the pose prediction learning flow even with occluded instances. Also, robust detector design must also contemplate adversarial attacks, which represent a high potential safety threat to pedestrians and other drivers. The survey did an extensive report on how 3 types of attacks affect 3D detection. They decided to apply disturbances to class labels, object position, and orientation, inject patch noises to 2D bounding boxes and dynamically resized them depending on the target size of the object. The findings of the work in showed that: depth-estimation-free approaches are more sensitive to adversarial attacks, BEV representation only provides robustness in class perturbation, and temporal integration or multi-view could be integrated into current networks to mitigate adversarial attacks even further.

It has also been observed that sensor fusion pipelines have gained considerable attention after attaining good positions in KITTI testing score ranking. These methods can handle occlusion since they fuse features from multiple inputs. For instance, if a stereo camera setup suffers from occlusion, LiDAR features may be enough to complement visual aspects and predict the bounding (Kim et al., 2023), the authors alleviated the gap between image feature representation and LiDAR point cloud by fusing these in a voxel feature volume to infer 3D structure of the scene. While the authors of (Wu et al., 2023) suggested a reduction in the redundancy of virtual point clouds and proposed an increase in depth accuracy by fusing RGB and LiDAR data in a new operator called, VirConv (Virtual Sparse Convolution) which is based on a transformed refinement scheme. Furthermore, to primarily rely on visual information, multi-sensor fusion can be done under a collaborative or networked 3D object detection pipeline under considerations of bandwidth and network schemes discussed in the previous section. Altogether, the discussed aspects in this section is poised to be an integral part of the research interests in the following years to accomplish reliable 3D object detection in autonomous driving.

## 6 Conclusion

This survey presented an in depth discussion regarding 3D object detection for autonomous driving using stereo and monocular cameras. At first, the 2D object detection techniques were covered and their challenges were highlighted in urban settings, which in turn motivates 3D object detection techniques in real-time. A classification composed of model- and learning-based was then presented to reflect the different feature extraction and 3D structure learning. This was subsequently followed by a detailed comparison of real-time capabilities for each approach, through its inference time (FPS) and KITTI dataset validation results. Furthermore, a discussion was provided on depth inference foundation, learning schemes, and internal representation based taxonomy with three classes: geometrically limited, end-to-end learning, and hybrid methods. Further, assessment indicators have been included to emphasise the benefits and shortcomings of each category of these techniques. To summarize, this paper aimed to provide a comprehensive survey and quantitative comparisons with state-of-the-art 3D object detection methodologies and identified research gaps and potential future directions in visual-based 3D object detection approaches for autonomous driving. On top of the identified research trends and challenges, the authors encourage to put detailed focus to the social implications of AI usage in aspects of policy making that addresses security and job concerns, eco-friendliness of AVs, economical impact, and availability of this resource in unrepresented social groups and countries.
